# Factors influencing kinesiophobia after radiofrequency catheter ablation in patients with atrial fibrillation: a cross-sectional study

**DOI:** 10.3389/fpubh.2026.1807261

**Published:** 2026-05-04

**Authors:** Yingying Zheng, Ruijia Xu, Mengying Yang, Hui Zhang, Taomei Zuo, Peipei Yu

**Affiliations:** 1 Department of Cardiovascular Medicine, The Central Hospital of Wuhan, Tongji Medical College, Huazhong University of Science and Technology, Wuhan, China; 2 Key Laboratory for Molecular Diagnosis of Hubei Province, The Central Hospital of Wuhan, Tongji Medical College, Huazhong University of Science and Technology, Wuhan, China

**Keywords:** atrial fibrillation, factors influencing, kinesiophobia, postoperative rehabilitation, radiofrequency catheter ablation

## Abstract

**Background:**

Kinesiophobia is a significant psychological factor that affects early ambulation in postoperative patients. It is particularly common after radiofrequency ablation for atrial fibrillation. Nonetheless, few studies comprehensively or systematically explore how physical, psychological, and disease-related objective factors predict kinesiophobia.

**Objective:**

The primary aim of this study is to examine the level of kinesiophobia and its contributing factors among patients following radiofrequency ablation for atrial fibrillation. This research aims to provide clinical evidence for the development of targeted intervention strategies to reduce motor phobia. These strategies aim to boost patients’ motivation and adherence to early physical activity, ultimately improving cardiac health post-surgery.

**Methods:**

A cross-sectional survey was conducted, assessing patients using questionnaires, including the Tampa Scale for Kinesiophobia Heart, Self-efficacy for exercise Scale, the Perceived Social Support Scale, the Fried Frailty Phenotype Scale, the Generalized Anxiety Disorder-7, the Patients’ Health Questionnaire Depression Scale, Physiological indicators related to atrial fibrillation, and echocardiographic examinations. Univariate and multivariate regression analyses were used to investigate the factors influencing kinesiophobia. The difference was considered statistically significant at *p* < 0.05.

**Results:**

The study included 356 patients. Multiple linear regression analysis identified the following influencing factors: frailty (β = 0.168: 95% CI, 0.606 ~ 1.375), LAD (β = 0.048: 95% CI, 0.007 ~ 0.077), LVEF (β = −0.117: 95% CI, −0.124 ~ −0.057), past history of stroke (β = 0.178: 95% CI, 1.919–4.126), combined heart failure (β = 0.182: 95% CI, 1.549–3.283), EHRA symptom classification (β = 0.121: 95% CI, 0.575–1.340), disease course (β = 0.075: 95% CI, 0.272–0.965), disease recurrences (β = 0.062: 95% CI, 0.023–1.843), perceived social support (β = −0.309: 95% CI, −0.225 ~ −0.137), and GAD-7 (β = 0.443: 95% CI, 0.651–0.939). These factors significantly influenced kinesiophobia in patients after radiofrequency ablation of atrial fibrillation (*p* < 0.05). They accounted for 86.4% of the total variance in kinesiophobia.

**Conclusion:**

Multiple factors influence kinesiophobia in patients after radiofrequency ablation for atrial fibrillation. Healthcare providers should prioritize risk assessment and individualized rehabilitation plans while fostering a support system that includes psychological care, family, and social resources to reduce kinesiophobia and support a safe resumption of daily activities.

## 1 Introduction

Atrial fibrillation (AF) represents the most prevalent persistent arrhythmia encountered in clinical practice. In recent years, the prevalence, incidence, and mortality associated with atrial fibrillation in China have shown a consistent upward trend (1, 2). The atrial fibrillation management guidelines endorse radiofrequency ablation as the first-line electrophysiological treatment modality (3). This approach effectively diminishes the burden of atrial fibrillation, sustains sinus rhythm, enhances cardiac function, and reduces mortality. Its clinical benefits surpass those of conventional pharmacological treatments (4). Nevertheless, the rates of recurrence and readmission following the procedure remain relatively high (5).

In recent years, cardiac rehabilitation programs that emphasize exercise have become part of managing atrial fibrillation. Studies show that exercise-based rehabilitation is safe and effective, improving cardiovascular health after radiofrequency ablation (3). These programs can enhance exercise endurance, lower resting heart rate, alleviate anxiety and depression, and improve overall quality of life (6). The European Association for Cardio-Thoracic Surgery also found that regular exercise is associated with a lower risk of atrial fibrillation recurrence (7). Although many benefits have been confirmed, the exercise participation rate of patients after radiofrequency ablation for atrial fibrillation is low, and their compliance is poor (1, 8).

Kinesiophobia was first used to assess the degree of musculoskeletal pain (9). Heart disease kinesiophobia is described as a fear of physical activity or exercise due to the fear that activity or exercise may worsen the condition and cause other adverse cardiovascular events. Bäck et al. (10) and Keessen et al. (11) found that cardiac rehabilitation compliance declines as patients’ fear of exercise increases. Multiple studies report that fear of movement affects 57–74% of patients with atrial fibrillation (8, 11, 12). This fear prevents patients from engaging in physical exercise. As a result, patients adopt passive avoidance behaviors, which decrease their activity levels and further lower their quality of life (13). Therefore, it is necessary to investigate the factors influencing Kinesiophobia in these patients to provide a basis for subsequently formulating targeted intervention strategies.

Although existing studies have explored the influencing factors of Kinesiophobia in patients after atrial fibrillation ablation. Ding et al. (8) and Li et al. (14) mainly analyzed demographic characteristics (age, educational level, income level) and disease-related factors (disease course, symptom classification), but rarely included biochemical indicators and objective examination data such as echocardiography. At present, there is still a lack of systematic research on developing a comprehensive model of influencing factors that integrates physiological, psychological, and social factors. Therefore, this study aims to comprehensively incorporate psychological, social, and physiological indicators related to atrial fibrillation, echocardiography-assisted examinations, and other factors, investigate the current status of kinesiophobia in patients after radiofrequency ablation of atrial fibrillation, and deeply analyze its influencing factors, with the aim of providing a basis for formulating targeted intervention strategies and reducing patients’ Kinesiophobia.

## 2 Methods

### 2.1 Design and study population

This is a cross-sectional study that utilized a convenience sampling method to select subjects. Patients diagnosed with atrial fibrillation who underwent radiofrequency ablation surgery in the Department of Cardiovascular Medicine at a tertiary hospital in Hubei Province between December 2024 and December 2025 were selected as participants. Statistical analysis was conducted on data from 356 patients. Inclusion criteria were as follows: (1) compliance with the diagnostic criteria for atrial fibrillation as outlined in the ESC Guidelines for the management of atrial fibrillation (15); (2) age of 18 years or older; (3) Three months after the completion of radiofrequency catheter ablation (RFCA), there were no serious postoperative complications; (4) provision of informed consent for participation in the study. Exclusion criteria included: (1) Those with a clear diagnosis of severe mental illness (such as schizophrenia, bipolar disorder, etc.), or those with cognitive impairment who are unable to complete the questionnaire; (2) contraindications for motor function tests.

### 2.2 Sample

In this study, the sample size was estimated based on the influencing factors related to kinesiophobia in atrial fibrillation Patients. According to the empirical formula for multivariate analysis sample-size estimation, sample size (*N*) = number of study factors (*n*) × (10~15). A total of 27 independent variables were included in this study, so the required sample size was calculated to be at least 270. A total of 380 questionnaires were subsequently distributed, and 356 valid questionnaires were recovered (effective recovery rate, 93.68%). The reasons for the 24 invalid/unrecovered questionnaires included: patient transfer to another hospital (*n* = 6); The questionnaire was incomplete or had logical errors (*n* = 10); Incorrect contact information or rejected calls (*n* = 8).

### 2.3 Data collection

This study was a cross-sectional descriptive study conducted on-site at an atrial fibrillation outpatient center 3 months after atrial fibrillation surgery. After the informed consent forms of the research participants were received, professionally trained researchers distributed questionnaires to the patients and explained their content, the purpose of the study, and how to complete the questionnaire. If any issues regarding the completion method or questionnaire content, the researcher would immediately return it to the participant for correction.

### 2.4 Measurements

#### 2.4.1 General information questionnaire

The general situation questionnaire was designed by the researchers based on previous studies and included demographic data. Age, gender, marital status, educational level, income, occupation, medical insurance; Disease-related information: Disease course, recurrence, past medical history, EHRA symptom classification, concomitant medication, physiological indicators related to atrial fibrillation (N-terminal pro-B-type Natriuretic Peptide, NT-proBNP), high-sensitivity cardiac troponin I, hs-cTnI. Echocardiography-assisted examinations (left ventricular ejection fraction, LVEF; left atrial diameter, LAD).

#### 2.4.2 Tampa scale for kinesiophobia heart (TSK-SV heart)

The scale, originally developed by Bäck et al. (16) was subsequently translated into Chinese. It comprises four dimensions: perception of danger (items 3, 8, 11, 16), fear of movement (items 2, 4, 12, 14, 17), avoidance of movement (items 1, 7, 9, 13), and dysfunction (items 5, 6, 10, 15). This scale uses a 4-point Likert scale, with items 4, 8, 12, and 16 reverse-scored, yielding a total possible score of 17–68. Higher scores indicate greater severity of motor phobia; a total score exceeding 37 points indicates a high level of motor phobia. The scale demonstrates strong internal consistency, as evidenced by a Cronbach’s alpha coefficient of 0.882. In this study, the scale demonstrated a Cronbach’s alpha coefficient of 0.828.

#### 2.4.3 Self-efficacy for exercise

This scale, originally developed by Resnick and Jenkins (17) and subsequently translated into Chinese by Lee et al. It consists of 9 items, each rated on a scale from 0 to 10. The scoring is based on the mean of these 9 items, with higher scores indicating greater exercise self-efficacy. The scale demonstrated a Cronbach’s alpha of 0.874. In this study, the scale demonstrated a Cronbach’s alpha coefficient of 0.923.

#### 2.4.4 Perceived Social Support Scale

This scale originally developed by Zimet et al. (18) and subsequently translated into Chinese by Jiang Ganjin. It consists of three dimensions: family, friends, and support from others, encompassing a total of 12 items. It employs a 7-point Likert scale, yielding a total score ranging from 0 to 84 score, with higher scores indicating a greater perceived level of social support. The scale demonstrates a Cronbach’s alpha coefficient of 0.896. In this study, the scale demonstrated a Cronbach’s alpha coefficient of 0.945.

#### 2.4.5 Fried frailty phenotype

The scale was proposed by Professor Fried in 2001 (19) and consists of five indicators: unexplained weight loss, self-reported fatigue, reduced physical activity, decreased walking speed, and low grip strength. Assessed through self-assessment questionnaires and objective measurements, respectively. The threshold values for physical strength, walking speed, and grip strength are stratified based on gender, height, and body mass index (BMI), respectively. This scale assigns 1 point for each frailty indicator met, with a total score of 0 to 5. A higher score indicates greater frailty severity. Chinese researchers have translated and applied this assessment tool into Chinese, demonstrating its scientific validity.

#### 2.4.6 Generalized Anxiety Disorder-7

This scale was developed by Spitzer et al. (20) in 2006 and subsequently translated into Chinese by He Xiaoyan. It comprises 7 items that assess factors such as tension and anxiety, the degree of worry, the extent of relaxation, and the ability to remain still, among others. The assessment uses a 4-point Likert scale, yielding a total score ranging from 0 to 21. Higher scores indicate greater severity of the patient’s anxiety condition. The scale demonstrates a Cronbach’s alpha coefficient of 0.898. In this study, the scale demonstrated a Cronbach’s alpha coefficient of 0.891.

#### 2.4.7 Patients’ Health Questionnaire Depression Scale-9

The scale, originally developed by Wang et al. (21), was subsequently translated into Chinese by Bian Cuidong. It comprises 9 questions and uses a 4-point Likert scale, yielding a total score range from 0 to 27. Higher scores indicate greater severity of the patient’s depression. The scale demonstrates a Cronbach’s alpha coefficient of 0.857. In this study, the scale demonstrated a Cronbach’s alpha coefficient of 0.815.

### 2.5 Ethical and research approvals

This study received approval from the Ethics Committee of Wuhan Central Hospital (Approval Number: WHZXKYL2024-266-01). All participants consented to participate in this study and provided written informed consent. All methods were carried out in accordance with relevant guidelines and regulations.

### 2.6 Statistical methods

Data entry and analysis were performed using SPSS Statistical 26.0. Measurement data were reported as M ± SD, and count data as frequencies and composition ratios (%). For non normally distributed measurement data were presented as median and quartile [P50 (P25, P75)]. To examine the influence of general information on exercise fear, *t*-tests and ANOVA were conducted. Next, correlations among frailty state, social support, psychology and exercise fear were assessed using Pearson’s correlation. Subsequently, variables that were significant in either the univariate or the correlation analyses were selected as independent variables (*p* < 0.05) with the TSK-SV Heart score as the dependent variable in multiple linear regression. Finally, *p* < 0.05 indicated statistical significance.

## 3 Results

### 3.1 Basic participant characteristics

The ages of the 356 patients ranged from 33 to 87 years; 49.16% (*n* = 175) were male, and 50.84% (*n* = 181) were female. A total of 76 patients (21.35%) had a primary school education or lower, 107 (30.06%) had a junior high school education, 111 (31.18%) had a high school education, and 62 (17.41%) had a college degree or higher. Approximately 3.93% (*n* = 14) of the patients had a monthly household income per person below ¥2,000. Table 1 shows the sociodemographic characteristics of study participants.

**Table 1:** General characteristics of after radiofrequency catheter ablation in patients with atrial fibrillation (*n* = 356).

Variables	*N* (%)	TSK-SV heart scores	*t/F*	*P*
Gender			0.718^a^	0.473
Male	175 (49.16)	43.39 ± 6.06		
Female	181 (50.84)	42.93 ± 5.88		
Domicile			−0.770^a^	0.938
City	344 (96.63)	(38.00 49.00)		
Rural area	12 (3.37)	(36.25 49.00)		
Occupation			0.791^b^	0.532
Retire	177 (49.72)	43.33 ± 5.89		
Peasant	21 (5.90)	43.52 ± 5.87		
Worker	22 (6.18)	42.91 ± 6.35		
Cadre	15 (4.21)	40.53 ± 6.11		
Other	121 (33.99)	43.21 ± 6.04		
Educational level			0.280^b^	0.840
Primary school and below	76 (21.35)	43.22 ± 5.77		
Middle school	107 (30.06)	43.47 ± 5.93		
Senior middle school	111 (31.18)	42.75 ± 5.89		
University	62 (17.41)	43.27 ± 6.48		
Personal monthly income (yuan)			0.062^b^	0.940
<2,000	14 (3.93)	43.14 ± 5.99		
2,000–4,000	192 (53.93)	43.06 ± 5.18		
>4,000	150 (42.14)	43.29 ± 6.19		
Payment method			0.022^a^	0.882
Private	16 (4.49)	43.38 ± 6.24		
Medical insurance	340 (95.51)	43.25 ± 5.97		
Smoking			−1.289^a^	0.198
Yes	66 (18.54)	42.30 ± 5.76		
No	290 (81.46)	43.35 ± 6.09		
Drinking alcohol			−0.250^a^	0.802
Yes	33 (9.27)	42.91 ± 6.26		
No	323 (90.73)	43.18 ± 5.95		
Concurrent hypertension			−0.380^a^	0.704
Yes	212 (59.55)	(38.00 49.00)		
No	144 (40.45)	(38.00 48.00)		
Concurrent diabetes mellitus			−0.732^a^	0.464
Yes	76 (21.35)	(38.00 50.00)		
No	280 (78.65)	(38.00 48.00)		
Concurrent coronary disease			−0.617^a^	0.537
Yes	122 (34.27)	(38.00 49.00)		
No	234 (65.73)	(38.00 49.00)		
Combined heart failure			20.737^a^	<**0.001**
Yes	100 (28.09)	50.22 ± 2.73		
No	256 (71.91)	40.40 ± 4.42		
Past history of stroke			13.961^a^	<**0.001**
Yes	51 (14.33)	51.84 ± 0.95		
No	305 (85.67)	41.70 ± 5.17		
Types of atrial fibrillation			−1.405^a^	0.161
Paroxysmal atrial fibrillation	220 (61.80)	42.82 ± 6.22		
Persistent atrial fibrillation	136 (38.20)	43.71 ± 5.51		
Disease course (years)			25.370^b^	<0.001
<3	103 (28.93)	41.4 ± 6.26		
3–5	169 (47.47)	42.45 ± 5.28		
>5	84 (23.60)	46.86 ± 5.35		
EHRA symptom classification			81.550^b^	<0.001
1	16 (4.50)	43.31 ± 6.83		
2a	189 (53.09)	39.81 ± 4.09		
2b	110 (30.90)	46.82 ± 4.42		
3	41 (11.51)	49.15 ± 6.08		
4	0 (0)	–		
Concurrent medications (types)			1.480^a^	0.140
<3	16 (4.49)	45.31 ± 5.72		
≧3	340 (95.51)	43.06 ± 5.97		
Disease recurrences			−3.353^a^	0.001
Yes	69 (19.38)	45.29 ± 6.02		
No	287 (80.62)	42.64 ± 5.85		

a *t* value.

b *F* value.

### 3.2 Univariate analysis of kinesiophobia after radiofrequency catheter ablation in patients with atrial fibrillation

The total kinesiophobia score in atrial fibrillation patients 3 months after surgery was (43.16 ± 5.97), with 71.63% at a high level of kinesiophobia, scores of each dimension: perception of danger (9.62 ± 1.76), fear of movement (12.04 ± 2.06), avoidance of movement (9.94 ± 1.66), and dysfunction (9.30 ± 2.01). The univariate analysis revealed statistically significant differences in kinesiophobia scores among patients with atrial fibrillation for past history of stroke, past history of heart failure, duration of atrial fibrillation, EHRA symptom classification, and recurrence status (*p* < 0.05; see Table 1). The above statistically significant factors were included in the multiple linear regression analysis.

### 3.3 Correlation analysis of kinesiophobia after radiofrequency catheter ablation in patients with atrial fibrillation

The findings from the correlation analysis indicated that fried frailty phenotype (FFP), generalized anxiety disorder-7 (GAD-7), patients’ health questionnaire depression scale-9 (PHQ-9), NT-proBNP, LAD, and hs-cTnI showed significant positive correlations with the level of kinesiophobia (*p* < 0.001). Conversely, self-efficacy for exercise (SEE), perceived social support scale (PSSS), and LVEF demonstrated significant negative correlations with the level of kinesiophobia (*p* < 0.001), see Table 2.

**Table 2:** Correlation analysis of kinesiophobia after radiofrequency catheter ablation in patients with atrial fibrillation.

Items	Scores	TSK-SV heart scores	
		*r*	*P*
FFP	(2.0 3.0)	0.819	<0.001
SEE	3.57 ± 0.95	−0.815	<0.001
PSSS	55.50 ± 0.54	−0.837	<0.001
GAD-7	4.52 ± 0.17	0.848	<0.001
PHQ-9	7.03 ± 0.19	0.734	<0.001
NT-proBNP	206.21 ± 9.80	0.768	<0.001
LEVF	58.12 ± 0.41	−0.234	<0.001
LAD	39.49 ± 0.36	0.117	0.014
hs-cTnI	(0.001 0.030)	0.872	<0.001

Fried frailty phenotype (FFP); Self-efficacy for exercise (SEE); Perceived Social Support Scale (PSSS); Generalized Anxiety Disorder-7 (GAD-7); Patients’ Health Questionnaire Depression Scale-9 (PHQ-9); N-terminal pro-B-type Natriuretic Peptide (NT-proBNP); high-sensitivity cardiac troponin I (hs-cTnI); left ventricular ejection fraction (LVEF); left atrial diameter (LAD).

### 3.4 Multiple linear regression analysis of the influencing factors kinesiophobia after radiofrequency catheter ablation in patients with atrial fibrillation

In the preliminary regression model, multicollinearity was detected for NT-proBNP (VIF = 6.068) and Self-efficacy for exercise (SEE) (VIF = 7.292), exceeding the conventional threshold of 5. PHQ-9 score, though marginally below the threshold (VIF = 4.968), was nonsignificant (*p* = 0.602) and conceptually overlapped with anxiety. To avoid bias in the estimation of regression coefficients and ensure model stability, this study eliminated the three variables listed above and refitted the model. With these adjustments completed, the VIF values of all independent variables in the final model were <5 (range: 1.068–2.886; tolerance > 0.4), indicating no multicollinearity and a stable, reliable model. Residual diagnosis shows that normality, homoscedasticity, independence, and linearity are all satisfied, and the Cook distance is less than 1. There are no strong influence points, and the regression hypothesis is valid. The final analysis results show that factors such as frailty, LAD, LVEF, Past history of stroke, Combined heart failure, EHRA symptom classification, Disease duration, Disease recurrences, Perceived Social Support, and anxiety significantly influenced kinesiophobia in patients after radiofrequency ablation of atrial fibrillation (*p* < 0.05). These factors accounted for 86.4% of the total variance in kinesiophobia (see Table 3).

**Table 3:** Multiple linear regression analysis of the influencing factors kinesiophobia after radiofrequency catheter ablation in patients with atrial fibrillation.

Variables	*B*	SE	β	95% CI		*t*	*P*	VIF
Constant	37.000	2.835	–	31.425	42.576	13.053	<0.001	–
FFP	0.991	0.195	0.168	0.606	1.375	5.072	<0.001	2.863
hs-cTnI	0.181	0.266	0.016	0.104	0.342	0.682	0.496	1.354
LEVF	−0.091	0.017	−0.117	−0.124	−0.057	−5.337	<0.001	1.252
LAD	0.042	0.018	0.048	0.007	0.077	2.386	0.018	1.068
EHRA symptom classification	0.957	0.194	0.121	0.575	1.340	4.924	<0.001	1.584
Past history of stroke	3.022	0.561	0.178	1.919	4.126	5.386	<0.001	2.801
Combined heart failure	2.416	0.441	0.182	1.549	3.283	5.479	<0.001	2.886
Disease course	0.619	0.176	0.075	0.272	0.965	3.513	<0.001	1.191
Disease recurrences	0.933	0.463	0.062	0.023	1.843	2.018	0.044	2.458
PSSS	−0.181	0.022	−0.309	−0.225	−0.137	−8.107	<0.001	2.788
GAD-7	0.795	0.073	0.433	0.651	0.939	10.848	<0.001	2.167

Fried frailty phenotype (FFP); high-sensitivity cardiac troponin I (hs-cTnI); left ventricular ejection fraction (LVEF); left atrial diameter (LAD); Perceived Social Support Scale (PSSS); Generalized Anxiety Disorder-7 (GAD-7).

## 4 Discussion

This study examined various indicators, including physiological, psychological, disease-specific, and social support factors, to determine the causes of kinesiophobia in patients with atrial fibrillation following radiofrequency ablation. The results show that fear of movement is more than just a psychological issue. Instead, it reflects an external sign of a multi-system functional imbalance.

### 4.1 The level of kinesiophobia in patients with atrial fibrillation after radiofrequency ablation

The total kinesiophobia score in atrial fibrillation patients 3 months after surgery was (43.16 ± 5.97), with 71.63% at a high level of kinesiophobia, similar to Knapik et al. (22) and Ding et al.’s (8) findings. The dimensional distribution pattern suggests that the early postoperative kinesiophobia is mainly manifested as a direct fear of movement itself and the possible adverse consequences it may cause, which in turn leads patients to actively avoid physical activities. The level of kinesiophobia can affect patients’ compliance with postoperative exercise rehabilitation, suggesting that, in clinical practice, the negative effects of exercise fear on patients after atrial fibrillation surgery should be emphasized (12, 14). Patients with a high level of exercise fear should be identified as early as possible, and systematic, scientific guidance should be provided to this group to effectively reduce their fear, as it might have a substantial role in improving patient outcomes and enhancing their recovery process, allowing for a smoother transition back to everyday life and activities.

### 4.2 Influencing factors of kinesiophobia in patients with atrial fibrillation after radiofrequency ablation

#### 4.2.1 Physiological factors

##### 4.2.1.1 Frailty

Frailty reflects a patient’s basic physiological state. It is marked by a decline in the functional reserves of multiple systems. Frailty shares risk factors and mechanisms with atrial fibrillation. These include aging, chronic diseases, excessive inflammation, and impaired autonomic nerve regulation of the heart (23, 24). This study shows that frailty is a positive predictor of patients’ kinesiophobia. Analyzing the reasons, one possible explanation is that a decrease in physiological reserves reduces exercise tolerance, making patients more prone to fatigue (25). Secondly, it may be related to the patient’s decreased muscle strength and increased balance disorders, thus worrying about falls caused by exercise (26, 27). Therefore, in clinical practice, frailty screening should be included in follow-up after atrial fibrillation surgery, and individualized exercise management should be tailored to the degree of frailty (28). At the same time, the popularization of frailty and exercise knowledge should be strengthened through various means, providing easy-to-understand exercise guidance and scientific information to reduce the fear of falls and promote moderate exercise, and to break the negative cycle of the interaction between physiology and psychology (24).

##### 4.2.1.2 The structure and function of the cardiac system

This study has shown that the LAD is positively correlated with kinesiophobia in patients after atrial fibrillation surgery. Conversely, LVEF was negatively correlated with kinesiophobia in patients following atrial fibrillation surgery. On the one hand, an increase in left atrial anteroposterior diameter is a key factor in the onset and progression of atrial fibrillation; Framingham’s study (29) reported that for every 5 mm increase in left atrial anteroposterior diameter, the risk of atrial fibrillation increases by 39%. On the other hand, left ventricular ejection fraction (LVEF) is a commonly used clinical indicator for evaluating cardiac pump function (30). Atrial fibrillation leads to structural changes in the left atrium and functional impairment, decreased myocardial contractility, and reduced ejection fraction through atrial electrical and structural remodeling (31). Reduced cardiac output leads to inadequate perfusion of surrounding tissues, which activates neurohumoral mechanisms. The sympathetic-adrenal medullary system releases large amounts of catecholamines, increasing heart rate and making patients more prone to shortness of breath and fatigue (31, 32). This not only reduces their exercise capacity but may also heighten their perception of exercise-related risks and exacerbate kinesiophobia. Cardiac structural and functional parameters provide an objective basis for assessing exercise-induced anxiety. Healthcare professionals can use these cardiac parameters to identify high-risk patients implement comprehensive management strategies, improve cardiac function, and lay the groundwork for exercise rehabilitation, but further research is needed to verify it.

#### 4.2.2 Disease characteristic factors

This study found that the related disease factors influencing the kinesiophobia in patients after atrial fibrillation surgery mainly include: past history of stroke, combined heart failure, disease recurrences, EHRA symptom classification, and disease course. Patients with a history of stroke, combined heart failure, or recurrence tend to have a higher degree of kinesiophobia, which is consistent with Zhang et al.’s (33) research results. Analyzing the reasons, it may be related to the weakness in the lower limbs, gait disorders, decreased attention, and limited activity left after a stroke (33). At the same time, inappropriate exercise is prone to induce symptoms such as tachycardia and breathing difficulties (34). Clinically, it is difficult for patients to distinguish the physiological cardiopulmonary responses caused by normal activities from the pathological somatic symptoms during the onset of heart failure and the recurrence of atrial fibrillation (13, 35). This may lead people to believe that exercise increases disease uncertainty, reduces self-efficacy, and eventually develops into kinesiophobia.

EHRA symptom classification and disease duration are important predictive factors influencing kinesiophobia, which is consistent with the research results of Knapik et al. (22). The analysis of the reasons reveals that the symptom classification and disease course are closely related to the severity of atrial fibrillation. Patients with higher symptom classification and longer disease course tend to have more severe conditions (7, 35). Most patients, out of concern about the risk of adverse cardiovascular events during exercise, adopt an evasive attitude toward it to avoid physical discomfort or more serious consequences, which seriously hinders their enthusiasm and initiative for exercise. Studies have shown that task-oriented balance training, such as gait training, sports games, yoga, and functional exercises, can enhance balance and reduce fear-avoidant behaviors (36). It is suggested that medical staff provide patients with fine-motor skills training. By predicting balance and lower limb strength, the activity ability of patients can be effectively improved (36), although there have been relevant studies, further verification is still needed in patients with atrial fibrillation. In addition, in clinical practice, nursing staff should carefully assess patients’ overall condition, identify early fear of exercise, and provide appropriate care. Strengthen health education to help patients distinguish between physiological and pathological somatic reactions. Based on the patient’s condition, cognitive readiness, and exercise preferences, provide targeted guidance to help them build confidence in exercise and eliminate negative emotions.

#### 4.2.3 Psychological and social factors

Anxiety is one of the influencing factors for kinesiophobia in patients after atrial fibrillation surgery. The patient’s level of fear of movement is positively correlated with anxiety, which was consistent with the findings of Bäck et al. (10). Farris et al.’s (37) research on patients’ anxiety sensitivity and avoidance of physical activity showed that for every one-point increase in the anxiety sensitivity score, the probability of patients avoiding physical activity increased by 4.5% Studies have shown that 27.6% of patients participating in cardiac rehabilitation exhibit significant anxiety (1). The reason is that patients with anxiety may be reluctant to persist in exercise rehabilitation due to irrational fears, such as a sense of vulnerability to life or the belief that exercise rehabilitation will exacerbate a poor prognosis. For instance, overestimating the risk of arrhythmia caused by exercise and overly focusing on changes in heart rate and physical sensations (38). For these patients with anxiety, medical staff should identify negative emotions such as anxiety as early as possible. Before participating in exercise rehabilitation, various intervention methods, such as mindfulness-based stress reduction therapy (39) and narrative nursing (40) were used can be adopted to alleviate patients’ anxiety and excessive alertness. Correct unreasonable beliefs and actively encourage them to engage in sports rehabilitation as soon as possible.

This study demonstrated that patients’kinesiophobia inversely correlates with their perceived social support, aligning with findings of Husheng et al. (14). Khan et al.’s (41) research indicated that social support, including encouragement and support from family members and peers, was a crucial factor enabling patients to actively engage in sports activities. Thompson et al.’s (42) study indicated that when facing health challenges, patients with similar illness experiences or similar ages can more easily establish trusting relationships. They tend to share their sports experiences and are willing to participate in physical activities together. In summary, patients with high social support can feel supported by peers, family, and healthcare professionals. This can provide important external motivation for patients with atrial fibrillation to engage in physical exercise, offer the necessary emotional and practical support, alleviate their kinesiophobia, and help them maintain a regular exercise routine and gradually develop healthy exercise habits (43). Healthcare providers can develop comprehensive intervention plans for patients who have undergone atrial fibrillation surgery and fully engage their social support networks. Encourage family members and caregivers to play an active role, increase their support and companionship for patients, and enhance patients’ perception of social support. In addition, peer support can help patients share successful experiences and practical strategies, thereby boosting their self-efficacy and alleviating their fear of physical exercise (42). In future research, experiments can be conducted to verify its positive impact on kinesiophobia in patients after atrial fibrillation surgery.

## 5 Conclusion

This study employs a cross-sectional design to thoroughly investigate the various influencing factors, including disease-related and psychosocial elements, on the kinesiophobia levels of patients with atrial fibrillation following radiofrequency ablation. Notably, individual heterogeneity in motor fear levels among these patients is evident. In clinical practice, healthcare professionals should accurately identify and implement tailored interventions tailored to each subtype. This approach aims to reduce exercise-related fear, thereby enhancing patient engagement in exercise rehabilitation and, subsequently, reducing the incidence of adverse cardiac events.

## 6 Strengths and limitations

In this study, not only were patient-reported outcomes included, but also reliable objective data were used to identify more specific and comprehensive influencing factors. Despite the study’s supportive findings, several limitations must be acknowledged. First, we included only AF patients from one hospital, and there was a lack of multicenter samples from different hospitals, resulting in limited sample representation and potentially non-generalizable results for all AF patients in China. Second, because we used a cross-sectional study, we could not draw valid causal conclusions; Furthermore, this study did not conduct a statistical analysis at the subdimensional level of the multi-dimensional scale, nor did it conduct a refined exploration of the relationship between it and kinesiophobia. Future research should adopt a more fine-grained dimensional analysis to reveal potential clinical associations. Finally, Although the relatively high R^2^ (86.4%) suggests that the selected variable accounts for a significant portion of the outcome variable’s variation, external validation is still required. It is recommended to conduct long-term, large-scale, multi-center collaborative research in the future to make the research results more representative and to provide a basis for the subsequent formulation of effective intervention plans.

## Data Availability

The original contributions presented in the study are included in the article/supplementary material, further inquiries can be directed to the corresponding author.
